# What has vision science taught us about functional MRI?

**DOI:** 10.1016/j.neuroimage.2022.119536

**Published:** 2022-08-03

**Authors:** Marc M. Himmelberg, Justin L. Gardner, Jonathan Winawer

**Affiliations:** a Department of Psychology, New York University, NY, USA; b Center for Neural Science, New York University, NY, USA; c Department of Psychology, Stanford University, CA, USA

## Abstract

In the domain of human neuroimaging, much attention has been paid to the question of whether and how the development of functional magnetic resonance imaging (fMRI) has advanced our scientific knowledge of the human brain. However, the opposite question is also important; how has our knowledge of the brain advanced our understanding of fMRI? Here, we discuss how and why scientific knowledge about the human and animal visual system has been used to answer fundamental questions about fMRI as a brain measurement tool and how these answers have contributed to scientific discoveries beyond vision science.

## 1. Introduction

The field of vision science, like other domains of cognitive neuroscience, has widely adopted functional MRI (fMRI) as one of its core tools. This has led some researchers to ask how much, if anything, fMRI has taught us about the human visual system. A symposium at the 2021 annual meeting of the Vision Sciences Society was dedicated to this question (Aguirre et al., 2021). Here, we draw attention to the fact that many vision scientists have used fMRI to answer the opposite question.

What has our existing knowledge of the visual system taught us about functional MRI?

fMRI’s potential as a tool for advancing our understanding of brain function depends on the properties of the tool and the signal that it measures. Here, we observe that vision science has been especially fruitful in characterising fMRI – both the instrument itself and the neurovascular signal underlying its measurements. This is because vision science allows one to control an input stimulus which predictably controls the neural signal in space and time. One can then link the expected neural signal to the observed fMRI signal, increasing our understanding of what fMRI is measuring. Such studies do not necessarily lead to a new understanding of how the visual system encodes information, nor should we expect them to. Rather, the goal is an improved understanding of fMRI and the blood oxygen-level dependent (BOLD) signal that is the basis of most fMRI measures. This, in turn, is useful for making new discoveries in other aspects of brain function where less is known about the relationship between input (stimulus or task) and neural responses. In particular, vision science has been used to address questions such as:

- What does fMRI measure?
- What is the nature of the hemodynamic response function (HRF)?
- What is the resolution of information that fMRI can measure?
- Can information within the fMRI signal be used in decoding and encoding models?
- Do the effects of large draining veins on the fMRI signal obscure our view of local neural activity?
- Are fMRI-based parcellations of the cortex reliable and are computational fMRI methods reproducible?

In the long run, scientists and clinicians are more interested in understanding brain function than the tools used to measure it. However, the former depends on the latter. Here we present a summary of research in which the systematic nature of vision science has been used to answer the above questions, and how these answers have contributed to scientific disciplines beyond vision.

## 2. What does fMRI measure?

Early fMRI studies exposed rodents to global physiological stimulation and showed that blood oxygenation can be used as an endogenous contrast agent for MRI (Ogawa et al., 1990a, 1990b). This suggested that fMRI might be used to indirectly measure neural activity. However, until experiments were conducted in humans with sensory stimulation, it was unknown exactly how neural activity would affect the BOLD signal. In fact, Ogawa and Colleagues (1990a) speculated that the BOLD signal might decrease during heightened neural activity: “*When some region in a brain is much more active than other regions, the active region could show darker lines in the image because of the increased level of deoxyhemoglobin resulting from higher oxygen consumption*”.

Within a couple of years, human fMRI measurements were made with visual (Kwong et al., 1992; Ogawa et al., 1992) and motor (Bandettini et al., 1992) stimulation and the opposite was found: Stimulation caused the fMRI signal to increase, consistent with foundational work from the 1980s showing that neural activity can lead to an increase in the supply of oxygenated blood outstripping the consumption of oxygen (Fox and Raichle, 1986). Ogawa et al. (1992) found that visual stimulation increased the water proton signal in primary visual cortex (V1). Importantly, they found that the proton signal in the tissue nearby (outside of) small vessels increased, and that this signal could be followed in time with the fMRI measurement. Shortening the echo time reduced the fMRI signal contrast, indicating that signal changes are caused by a change in T_2_ * relaxation. This was the first human study to show that a stimulus drives an intrinsic contrast agent (changes in the concentration of deoxyhemoglobin in cerebral blood) and that this is what BOLD measures. This study used visual stimulation because the researchers knew precisely where in the cortex to look for responses and because they knew that a visual stimulus would produce strong neural activity that could be easily controlled. It would have made little sense to attempt to make new discoveries about the neural basis of perception while simultaneously trying to learn how fMRI works.

As implied from the initial fMRI work, the BOLD signal depends on a complex interplay of changes in oxygen consumption, blood flow, and blood volume. Vision science has been used to understand the interplay of these processes and how they are reflected in the fMRI response. In particular, much foundational work on the biological contributions to the BOLD signal have depended on experiments with visual stimulation, including fundamental work on glucose (Fox et al., 1988) and oxidative metabolism (Hoge et al., 1999; Thompson et al., 2004, 2003) in the human brain; on calibrated fMRI (Blockley et al., 2013; Davis et al., 1998); on the contribution of cerebral metabolic rate of oxygen consumption, blood flow and volume to the BOLD response (Buxton et al., 2004; Kwong et al., 1992; Lu et al., 2003; Ogawa et al., 1993) and on the spatial localization of these components (Duong et al., 2001; Malonek and Grinvald, 1996; Vanzetta and Grinvald, 1999; Zhao et al., 2006). These studies used visual stimulation because it provided a specific cortical target (calcarine sulcus), it enabled precise temporal control of neural activity, and it induced large, sustained neural activity as long as the stimulus is present (for review see Hillman 2014).

These key studies linked blood oxygenation to the fMRI response; however, they did not address what kinds of neural activity might cause changes in blood oxygenation. It is likely that many facets of neural activity influence the BOLD signal and there is not a single, simple answer to this question (and we do not attempt to answer it here). For reviews targeting this question, see Drew (2019), Logothetis (2003), and Logothetis and Wandell (2004). However, vision science found a useful way to reframe and address this question by using known properties of the visual system in specific brain areas and well-oiled stimulus regimes.

Visual neurophysiology experiments in animal models have described neural responses to systematic variations in fundamental visual properties, such as image contrast and motion coherence, well before the advent of fMRI. Researchers were able to take advantage of these neural responses to better understand the underlying neural signal that fMRI measures. Here, we focus on examples of research linking the fMRI signal to stimulus manipulations for which the neural response was known prior to the fMRI experiments. Work from Rees et al. (2000) used visual area hMT+ to explore the neural basis of the fMRI signal. The signal in hMT+ increased linearly with stimulus motion coherence (cf. Birman and Gardner 2018), as did prior measurements of average single neuron firing rates in monkey MT, linking the two signals together. Support came from a follow-up from Heeger et al. (2000) who compared the fMRI signal in human V1 with electrophysiological recordings of neural firing from monkey V1. Both measurements were driven by stimuli that systematically varied in contrast. Moreover, there was a proportional relationship between fMRI signal and the firing rate of V1 neurons. This link between the contrast response measured with electrophysiological and fMRI signals has been strengthened by experiments showing that characteristics such as adaptation (Gardner et al., 2005) and saturation (Vinke et al., 2022) can be reliably measured with fMRI. Moreover, simultaneous measurements of contrast response have shown a linear (Cardoso et al., 2012) or threshold-linear (Logothetis et al., 2001) relationship between the fMRI and electrophysiological signal, thereby forming a foundation for the linkage of these measured fMRI signals to behavioral measurements (Boynton et al., 1999).

Winawer et al. (2013) investigated the relation between the fMRI BOLD signal and electrocorticographic (ECoG) responses in visual cortex in the spatial domain. BOLD and broadband ECoG responses had the same sub-additive spatial summation, whereas stimulus locked ECoG responses did not. They concluded that asynchronous broadband signals (closely correlated with spiking) are an important contributing factor to the BOLD signal. Other work has used carefully controlled stimuli and a neural model to confirm that the broadband response in ECoG is well matched to the BOLD signal, but that additional variance in the BOLD signal is related to the power of low frequency oscillations (Hermes et al., 2019). This supports the claim that the BOLD signal is influenced by neural signals other than spiking (Logothetis and Wandell, 2004).

**Fig. 1.**
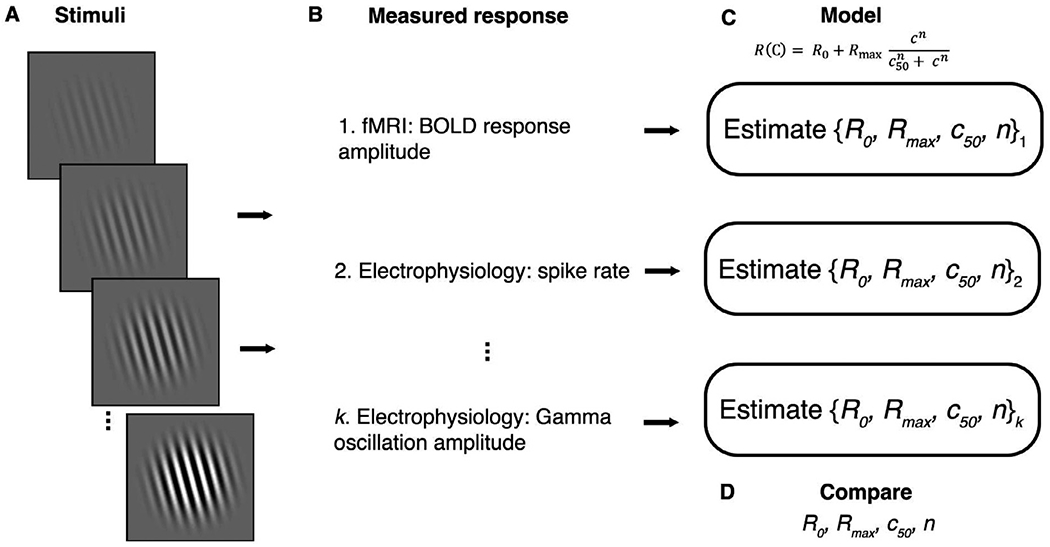
The neural basis of the fMRI signal can be tested by comparing responses with reference to parametric variations of an input stimulus. (A) A set of stimuli are chosen that parametrically vary in some dimension - in this case, spatial contrast. (B) Measurements are made in multiple modalities. These measurements do not need to be made simultaneously or even in the same individuals or species. (C) Responses are modeled as a function of the stimulus using the same model form (but different fitted parameters) for each modality. For example, one can estimate *R_0_*, *R_max_*, *c_50_*, and *n* for different measurement types in response to variations in stimulus contrast. (D) The model parameters are compared between multiple measurement types with reference to parametric variations in stimulus contrast. Note that the measurements in (B) are not directly compared to each other.

It was not necessary to complete simultaneous measurements in these studies because a quantitative link was enabled by matching stimulus parameters and recording locations. These studies shed light on the neural basis of the fMRI signal not by directly comparing the fMRI response to neural activity (as the two are measured in different units, time scales, and spatial extents) but by comparing each of them with reference to parametric variations in the visual stimulus (Fig. 1). Three advantages of this model-based, or ‘stimulus-referred’ method over a correlation method are that: (1) it is robust to variation in signal-to-noise across measurement modalities; (2) it ensures a large dynamic range in the responses; and (3) it captures responses to stimulus properties that are presumed to be important for information encoding. Finally, these stimulus-referred approaches have recently been expanded to ‘image computable’ approaches (Kay et al., 2008) that can provide deeper understanding of how responses at the neural level translate to population responses measured by fMRI (Gardner and Merriam, 2021).

## 3. What is the nature of the hemodynamic response function (HRF)?

**Fig. 2.**
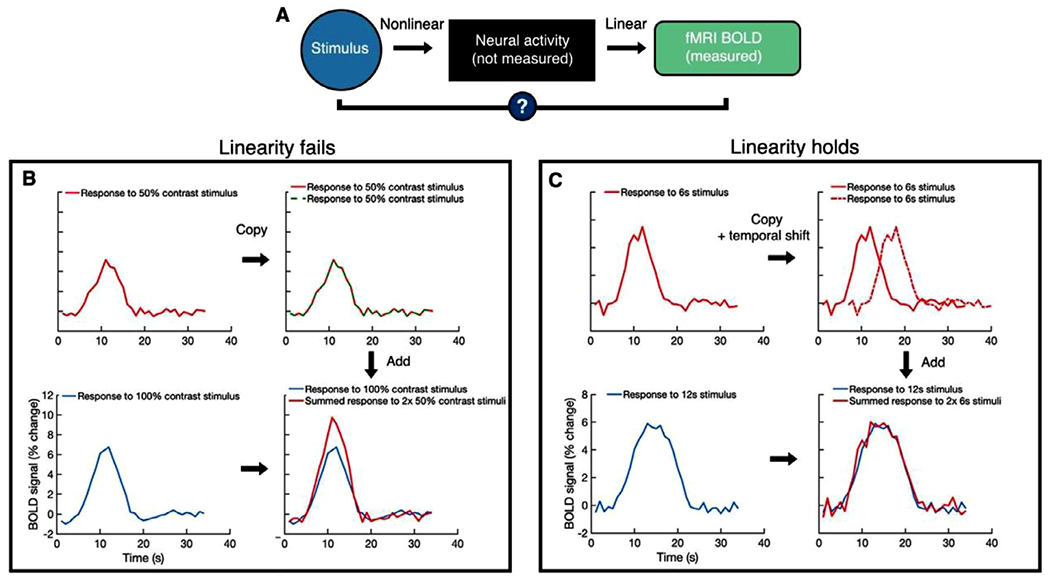
Testing the linear transform model of the fMRI response. (A) Neural activity tends to be a nonlinear function of the stimulus. The linear transform model tests whether the fMRI response is a linear function of neural activity. (B) Although the fMRI response monotonically increases with stimulus contrast, additivity fails. The summed fMRI response to 2 × 50% contrast stimuli is smaller than the response to 1 × 100% contrast stimulus. This is presumed to be due to non-linearities in the stimulus-to-neural transform. (C) The fMRI response obeys temporal additivity; the summed fMRI response to 2 × 6s stimuli shifted in time is similar to the response of 1 × 12s stimulus. Here, the neural response to the second 6 s period is assumed to be similar to the neural response to the first 6 s period. Note that BOLD signal in (B) and (C) is simulated.

Vision science has been used to assess whether the fMRI signal obeys linearity. Many fMRI analyses and experimental designs (especially fast, event-related designs) rely on the assumptions that the fMRI signal can be averaged across trials and sums approximately linearly in time. Boynton et al. (1996) tested a ‘linear transform model’ of the fMRI signal (Bandettini et al., 1993; Friston et al., 1995) in which V1 neural activity is a nonlinear function of stimulus contrast and the corresponding fMRI signal is a linear transform of this neural activity (Fig. 2A). In particular, the authors were interested in testing whether the fMRI response could be approximated as a shift-invariant linear transform of the neural response (averaged over local spatial and temporal extents). This was an important test, because if the answer is yes, one can measure the fMRI signal in many kinds of experiments, deconvolve it, and infer the neural responses to stimuli or tasks.

Testing linearity was an ambitious goal since the authors did not have direct access to the neural response and because the relationship between a stimulus and its BOLD response is presumed to contain non-linearities (as opposed to the neural response and the BOLD response, which may be approximately linear). The key to testing the linearity of the neural to BOLD transform was choosing and varying stimulus dimensions for which the neural response was expected to be linear. This was made possible from prior work in visual neuroscience. When they tested linearity by varying contrast (the ‘scaling’ property of a linear system), linearity failed (Fig. 2B). This was expected because the neural response to stimulus contrast is non-linear. When they tested temporal linearity at the scale of several seconds, linearity held: the response to a 12 s stimulus was well predicted by copying, shifting, and summing the response to a six-second stimulus (Fig. 2C). One caveat is that the brief stimuli gave a larger than expected response predicted by this linearity, likely due to neural adaptation (Boynton et al., 2012).

The linear transform model was more fully tested by convolving the predicted neural time-course with a shift-invariant linear temporal filter to predict the stimulus-evoked fMRI responses (Boynton et al., 2012, 1996). This was found to be a good fit. Thus, the hemodynamic response function (HRF) is approximately linear in time. The authors conducted this work in V1 because they required a region where they could localise the fMRI signal and because they understood how a visual stimulus would drive the neural –but not fMRI– response (Boynton et al., 2012).

Further, Dale and Buckner (1997) investigated whether selective averaging techniques could be applied to visually evoked fMRI responses. They found that the fMRI signal can be linearly summed across both short and intermixed trials. These two studies used simple contrast patterns of varying duration, and acted as the catalyst of the development of canonical HRF models that act as a transfer function between neural activity and the fMRI signal throughout the human brain (Huettel, 2012), laying the basis for thousands of subsequent event-related fMRI studies. This work also allowed researchers to use fMRI to investigate dimensions and brain regions for which there is no model of the neural response. However, for this work to take place, the nature of the fMRI signal itself had to be first established.

Likewise, vision science has contributed to our understanding of contributing factors to ‘negative BOLD’, that is, a decrease in the BOLD signal during experimental tasks. Shmuel et al. (2002) addressed a fundamental question in neuroimaging: does a negative BOLD response imply a reduction in neural activity or is it a purely vascular phenomenon (Wade, 2002)? They answered this by characterising negative BOLD in human V1-V3. Stimulus-contrast and stimulus-duration dependent changes in positive BOLD were mirrored in negative BOLD. To establish that the BOLD signal was negative, the authors defined a meaningful baseline as the BOLD response to a uniform field (mean luminance). They justified this choice based on classic vision science findings from Hubel and Wiesel (1962) demonstrating that the responses of neurons in early visual cortex are largely insensitive to mean luminance, driven instead by contrast. To probe the coupling between positive and negative BOLD, Schumel et al. (2002) interleaved fMRI BOLD scans and scans that measured cerebral blood flow. Clusters of negative BOLD were spatially correlated with reductions in cerebral blood flow, indicating that negative BOLD is due to a decrease in the rate of oxygen consumption, reflecting a decrease in neural activity in response to neural suppression. The locations of positive and negative BOLD on the cortical surface, combined with stimulus selection, enabled the researchers to interpret the results in terms of neural receptive fields (surround suppression). This work found support in a follow-up study, where Shmuel et al. (2006) showed that negative BOLD is associated with local decreases in neural activity measured from electrophysiology. Although negative BOLD does not always imply a decrease in neural activity, the value in these studies was a demonstration that negative BOLD can be caused by a decrease in neural activity. The importance was in providing a new characterisation of one part of the fMRI signal, rather than a discovery of how visual circuits work. Negative BOLD has become an increasingly important topic of investigation outside of visual areas, including resting state networks (Parker and Razlighi, 2019; Sestieri et al., 2011; Sormaz et al., 2018) and task-related responses in motor cortex (Yuan et al., 2011; Zeharia et al., 2012).

## 4. What is the resolution of information that fMRI can measure?

The organisation of visual regions into spatial maps enables estimation of the point-spread or line-spread function–the spatial extent of activation on cortex from a small stimulus. In V1, the line spread function (full width at half max) has been estimated to be about 3.5 mm (Engel et al., 1997). To determine whether fMRI can resolve neural activity at an even finer scale than the line function, the spatial pattern of neural activity must be precisely tailored; one cannot test the resolution limits of the BOLD signal if the neural activity is correlated across a large region of cortex. Vision science provided the theory on how to do this.

Ocular dominance columns in V1 were identified in animal models long before their initial measurement using fMRI (Horton and Hocking, 1996; Hubel and Wiesel, 1963a; Wiesel and Hubel, 1963). It was already known that human ocular dominance columns are ~1 mm wide (Adams et al., 2007; Adams and Horton, 2009) and each column’s ocular selectivity varies at a fine scale. Thus, ocular dominance columns were an ideal model for investigating the spatial resolvability of the fMRI signal, which may be limited by vascular blurring. Indeed, fMRI signals driven by visual input to the left or right eye could be reliably resolved by some fMRI sequences (Cheng et al., 2001; Yacoub et al., 2007), confirming the submillimeter resolvability of the fMRI signal.

This work showed that fMRI can be sensitive to fine-scale neural properties, enabling researchers to investigate functional subdivisions in the cortex at a high resolution. For example, classical electrophysiological work in non-human primates identified thin and thick stripes in V2 (Livingstone and Hubel, 1982; Roe and Ts’o, 1995; Tootell et al., 1983). These stripes are selective for colour (Hubel and Livingstone, 1985; Tootell et al., 2004) and binocular disparity (Hubel and Livingstone, 1987), respectively, and are ~1.3 mm wide in macaque (Tootell and Hamilton, 1989) –below the 3.5 mm line spread function of the BOLD signal. There has been uncertainty around the existence of these stripes in human V2. Indeed, 7T fMRI work has shown that human V2 also has a striped architecture (Dumoulin et al., 2017; Nasr et al., 2016), supporting the submillimeter resolvability of fMRI. These studies activated stripes by controlling specific stimulus properties (high vs low temporal frequency, achromatic vs chromatic, and with or without binocular disparity).

Together, these studies used known properties of the visual pathways (eye-of-origin selectivity, temporal and chromatic sensitivity within the magno- and parvocellular pathways) to target fine-scale structures. Thus, with careful analysis methods and tight stimulus control, the fMRI signal can be sensitive to fine scale neural properties. Understanding the spatial resolution of the fMRI signal is important for researchers seeking to make discoveries about the detailed organisation of cortical areas where functional subdivisions are uncertain–such as memory areas (Dalton et al., 2018; Doeller et al., 2010; Hodgetts et al., 2017) – or even unknown, such as language areas (Binder et al., 1997) for which animal model homologues do not exist.

## 5. Can information within the fMRI signal be used in decoding and encoding models?

Vision science has also been used to assess whether the fMRI signal can be linked to the representation of information in different brain areas. Popular fMRI classification and pattern-analysis techniques were first developed using vision experiments. Multivoxel pattern analysis (MVPA), which uses classification algorithms to search for patterns of fMRI activity across pools of voxels (see Norman et al. 2006), was developed using fMRI responses to faces, objects, and grating orientation in visual cortex (Haxby et al., 2001; Kamitani and Tong, 2005). Similarly, representational similarity analysis (RSA), which characterises neural representations of experimental conditions via the dissimilarity of fMRI activity patterns, was developed using fMRI responses to categorical visual object representations in ventral temporal cortex (Edelman et al., 1998; Kriegeskorte et al., 2008). These classification techniques have since been used to answer questions about topics outside of vision in regions beyond visual cortex, including spatial representation (Berens et al., 2021; Hassabis et al., 2009) and episodic memory (Chadwick et al., 2010) in the hippocampus, mnemonic representations in working memory (Kwak and Curtis, 2022), the representation of perceived body size in extrastriate body area (Carey et al., 2019), and the neural representation of emotion in brain regions associated with theory of mind (Skerry and Saxe, 2015).

The MVPA method has led to an active debate regarding the spatial scale of information driving successful pattern classification. Vision science has been central in this debate. Electrophysiological work in animal models identified orientation pinwheels in V1 in the form of orientation selective hypercolumns with a periodicity of ~2 mm (Blasdel and Salama, 1986; Hubel and Wiesel, 1963b; Ohki et al., 2006). Some have used these pattern classification analyses to seemingly decode orientation information from these fine-scale pinwheels (Alink et al., 2013; Haynes and Rees, 2005; Kamitani and Tong, 2005; Kay et al., 2008). However, others have argued that the decoding measurements are dominated by coarse-scale orientation biases (Freeman et al., 2013), rather than fine-scale activity. More recently, coarse-scale orientation biases have been linked to changes in the fMRI signal due to stimulus vignetting (i.e., the change in contrast along a stimulus edge) rather than cortical structure (Roth et al., 2018). These findings suggest pattern-classification techniques applied to other domains are also likely to be most sensitive to large-scale biases, rather than sub-millimetre structures. A practical lesson from this debate: when classification methods lead to accurate decoding in a novel paradigm, researchers ought to check for the existence of neural tuning at a coarse spatial scale (Gardner and Merriam, 2021).

These classification methods test the ability to decode inputs such as a visual stimulus. Vision science has also been at the forefront of developing encoding models tested with fMRI. These computational approaches successfully linked the fMRI signal to neural properties. For example, the population receptive field (pRF) model (Dumoulin and Wandell, 2008) provides a quantitative framework to link the fMRI signal with neural response properties of cortical cells. This framework is the genesis of many computational approaches to fMRI. The pRF model is defined in terms of input parameters that are informed by theory of visual receptive fields in visual cortex. Since its inception and initial application, the pRF model has been used to understand topographic organisation for other stimulus types and cortical regions: somatosensory cortex (Puckett et al., 2020; Schellekens et al., 2021; Wang et al., 2021), auditory cortex (Thomas et al., 2015), numerosity maps in parietal cortex (Harvey et al., 2013; Harvey and Dumoulin, 2017; van Dijk et al., 2021), sensory substitution (Hofstetter et al., 2021), semantic space (Huth et al., 2012), and event timing (Harvey et al., 2020). Further, the computational approach to studying visual cortex has demonstrated that the magnitude of the fMRI response, which is measured at the scale of seconds, is impacted by neural dynamics at the millisecond scale (Horiguchi et al., 2009; Stigliani et al., 2017; Zhou et al., 2017).

Having a domain like vision science, in which some of the results are expected from prior knowledge, has provided a solid foundation for the extension of the computational approach to other domains; for example, the pRF model has been expanded to assess the canonical computation of normalisation that is thought to occur throughout the brain (Aqil et al., 2021). Overall, the forward modeling approach provides an alternative to the subtraction approach (i.e., measuring contrast maps between stimuli, task, or groups) (Van Orden and Paap, 1997), affording greater generalisation and explanatory depth.

## 6. Do the effects of large draining veins on the fMRI signal obscure our view of local neural activity?

Artefacts in the fMRI signal can have vascular origins. Vascular draining can contaminate fMRI signal from any region of the cortex in which large veins exist, posing a fundamental problem of interpretation of the fMRI signal: “*The realization in 1993 of the large vein contribution was highly disturbing to us. Large veins drain blood from large patches of cortex and their distribution is spatially sparse. Therefore, they cannot provide high spatial fidelity to neuronal activity in functional imaging*” (Menon et al., 1993; Uğurbil, 2018). Understanding this complication for the entire field of fMRI was best addressed by harnessing known properties of neural circuits in visual cortex. Vision science enables specific predictions about expected fMRI responses, including their location, strength, and the timing of their activation. Thus, visual stimulation is well-suited to detect anomalous responses and then link these responses to vascular artefacts (e.g., Lee et al. 1995 and Winawer et al. 2010).

Visual experiments have been used to clarify the ability of fMRI to distinguish neural effects from vascular confounds. Kay et al. (2019) used simple visual stimuli to examine the relationship between veins and the fMRI signal in early visual cortex. The presence of veins amplified and caused spatial displacement of the fMRI signal. Likewise, vision science has contributed to the development of techniques that correct for venous artefacts. Kay et al. (2020) developed a method that produced data-driven estimates of venous effects on the fMRI signal. These effects were modeled and used to separate the fMRI signal into one component related to the microvasculature (capillaries and small venules) and one related to the macrovasculature (large veins). Olman et al. (2007) showed that a differential experimental design (rather than single stimulus condition interleaved with a blank baseline) minimises the contribution of large veins to the fMRI signal. Both techniques were developed using vision science experiments because the authors could spatially localise a robust fMRI signal. Finally, vision science has been used to validate the ability of spine-cho sequences to compensate for venous artefacts from large veins. Olman et al. (2012) used spin-echo sequences to identify fine-scale structures in visual cortex that would otherwise be masked by venous artefacts appearing in a gradient-echo sequence (Ugurbil, 2016). The general findings are that while vessel-related limits are certainly real, under appropriate conditions they can be corrected, and fMRI can reliably probe neural function at the millimetre scale.

The advent of 7T fMRI has given rise to the study of laminar circuitry in the human brain. Vision science has inspired models of how veins contribute to changes in the fMRI signal across cortical depth. The BOLD signal blurs towards the superficial surface due to ascending veins and surface vasculature (Duvernoy, 1999; Polimeni et al., 2010), causing a greater BOLD towards the superficial surface (Kay et al., 2019). However, this does not accurately reflect the distribution of neural activity. Havlicek and Uludağ (2020) modeled the effects of veins on the BOLD signal across lamina; depth-dependent variability in the BOLD signal originated from depth-dependent changes in vasculature. Importantly, their model was motivated by experimental observations about how the neural signal changes across lamina in animal visual cortex.

Correcting vascular artefacts is vital for achieving high resolution fMRI measurements. Results from past vision experiments have informed and validated methods that correct for venous artefacts across depth. Work from Markuerkiaga et al. (2021) found that deconvolving lamina activation profiles with a physiological point spread function removes venous artefacts (i.e., deconvolution ’flattens’ the trend of BOLD increasing towards the superficial surface). The point spread function was derived from a model of vasculature based upon histological studies of V1 (Markuerkiaga et al., 2016). Further, the deconvolved lamina profiles were validated via comparison to ’gold-standard’ profiles measured from human visual cortex (Fracasso et al., 2018). Understanding how the fMRI signal changes with cortical depth has opened new doors for research to investigate neural tuning properties (De Martino et al., 2015; Fracasso et al., 2016; Olman et al., 2012) and feed-back signalling (Klein et al., 2018; Kok et al., 2016; Lawrence et al., 2019; Muckli et al., 2015; Sharoh et al., 2019) across laminae.

## 7. Are fMRI-based parcellations of the cortex reliable and are computational fMRI methods reproducible?

One benefit of fMRI over other methods that probe brain function is its large field of view: one can sample the fMRI signal across the whole brain every second or so. Thus, fMRI can be used to understand how the brain is parcellated into discrete areas. Parcellation schemes are useful for understanding brain function and linking results across studies and laboratories. However, the utility of parcellations depends on their accuracy. Vision science has provided tools for validating parcellation schemes by comparing them against boundaries from retinotopic maps. If a parcellation scheme differs from known retinotopic maps, researchers can question the validity of the scheme.

For example, Laumann et al. (2015) used resting state functional connectivity to parcellate a highly scanned individual’s cortex. The parcellations were validated via their correspondence with measured retinotopic maps, especially V1-V3, as their borders are well-defined by polar angle reversals. The V1 parcellation aligned to its retinotopic boundary. However, cross-subject averaging of the parcellations resulted in false positives (parcellation boundaries that did not correspond to any retinotopic boundaries) and false negatives (retinotopic boundaries that did not correspond to any parcellation boundaries) in V2 and V3. Thus, cross-subject transformations of fMRI data obscured patterns in interindividual brain organisation, highlighting the importance of individual analysis of fMRI data. In a similar vein, Glasser et al. (2016) used a semi-automated neuroanatomical approach to parcellate group-level multimodal data from the Human Connectome Project (HCP). In this case, the parcellations of early visual field maps aligned to the retinotopically defined V1-V3 boundaries from Abdollahi et al. (2014), validating the accuracy of the parcellations and their multimodal method.

The reliability and reproducibility of fMRI methods have been usefully assessed with vision science methods. Recently, much attention has been placed on the reproducibility of psychology (Open Science Collaboration, 2015) and neuroimaging studies (Botvinik-Nezer et al., 2020; Marek et al., 2022; Poldrack et al., 2017). Human retinotopic maps are highly reproducible (Benson et al., 2018; Himmelberg et al., 2021; Lage-Castellanos et al., 2020; Lerma-Usabiaga et al., 2020; van Dijk et al., 2016) and large, publicly available datasets of fMRI responses in visual cortex, such as the HCP Retinotopy (Benson et al., 2018) and NSD datasets (Allen et al., 2022), are at the forefront of understanding brain function. Even the large sample sizes of retinotopic mapping datasets (Benson et al., 2018; Himmelberg et al., 2021) are relatively small when compared to the sample sizes needed for reproducibility of some fMRI methods (Marek et al., 2022), consistent with the idea that the fMRI response can be highly reliable when coupled to appropriate stimuli and analysis methods (Rosenberg and Finn, 2022). This high level of reproducibility in retinotopic data is due to the implementation of an explicit computational approach in characterising the fMRI signal.

## 8. Why has vision science been so useful for fMRI?

The methods underlying vision science guide us on how to drive the system with large signals that are spatially and temporally precise. For example, established knowledge of visual processing tells us that spatial and temporal contrast are more important stimulus parameters than luminance, and these parameters will drive the largest fMRI signal. One probably would not want to use, for example, language or emotion, as a tool to test the linear transform model of the BOLD response, as there may be unknown non-linearities in the stimulus-to-neural responses, and experimenters cannot precisely control the onset, offset, and intensity of the neural responses via their stimulus. On the other hand, one might apply the linearity findings from visual neuroimaging to help model the responses in a study of language or emotion. Likewise, the organisation of the visual system is well-documented, allowing for highly accurate spatial localisation of the fMRI signal in space and time. Finally, vision science equips us with tools to parametrically manipulate the strength of the neural and fMRI signal. For example, we know that contrast is the currency of the visual system and we understand how varying the contrast of a stimulus will drive both neural and fMRI signals. This allows researchers to define the fMRI response in units of visual stimulus and compare the fMRI response with measurements from other instruments.

We do not wish to argue that other fields should necessarily adopt the same methods described here, nor do we suggest that vision science has been the sole contributor to understanding fMRI. Indeed, disciplines beyond vision science have made major contributions to understanding fMRI. For example, one of the first human BOLD experiments targeted the motor system (Bandettini et al., 1992) and an important study on the neural basis of the BOLD signal used auditory stimuli (Mukamel et al., 2005), though the ease with which one can present calibrated stimuli and the precision with which we can predict the neural response are the appeal of using vision. Most of the studies we have described took advantage of known features of the nervous system to make discoveries about fMRI. When the goal is to make new discoveries about the nervous system rather than about fMRI, sometimes different methods are needed. Our point is instead that studies which characterise the measurement itself (fMRI and the BOLD signal) can be of great value, and that the tools of vision science are well suited to this goal.

The systematic (and perhaps tedious) nature of vision science has paid off; it has advanced our understanding of fMRI, starting with the BOLD signal and more recently with the development of computational models to characterise the fMRI response. Although we have focused on fMRI, a similar approach can be used to better understand other forms of brain measurement technology, such as functional ultrasound (Macé et al., 2011) or portable modular quantum magnetometer systems (Tierney et al., 2019). Overall, the advancement in our understanding of fMRI afforded through vision science has benefited psychology, by allowing psychologists to non-invasively measure the neural basis of a whole array of human behaviours and thereby shaping the way we think about human psychology, and medicine, by allowing medical researchers to detect changes in cortical neural circuit functioning in response to disease or therapy.

## Data Availability

No data was used for the research described in the article.
